# Duration of Diabetes Predicts Aortic Pulse Wave Velocity and Vascular Events in Alström Syndrome

**DOI:** 10.1210/jc.2015-1577

**Published:** 2015-06-11

**Authors:** Richard B. Paisey, Jamie Smith, Catherine Carey, Timothy Barrett, Fiona Campbell, Pietro Maffei, Jan D. Marshall, Christopher Paisey, Richard P. Steeds, Nicola C. Edwards, Susan Bunce, Tarekegn Geberhiwot

**Affiliations:** Diabetes Research Unit (R.B.P., J.S., C.C., S.B.), Horizon Centre, Torbay Hospital NHS Foundation Trust, Lawes Bridge, Torquay, Devon TQ2 7AA, United Kingdom; School of Clinical and Experimental Medicine (T.B.), College of Medicine and Dentistry, University of Birmingham, Edgbaston, Birmingham B15 2TT, United Kingdom; Leeds Children's Hospital (F.C.), Leeds, West Yorkshire LS1 3EX, United Kingdom; Internal Medicine 3 (P.M.), Department of Medicine, University Hospital of Padua, 35122 Padua, Italy; The Jackson Laboratory (J.D.M.), Bar Harbor, Maine 04609; University of Nottingham Medical School (C.P.), Nottingham NG7 2UH, United Kingdom; and Department of Cardiology (R.P.S., N.C.E.), and Department of Endocrinology (T.G.), University Hospitals Birmingham NHS Foundation Trust, Queen Elizabeth Hospital, Queen Elizabeth Medical Centre, Birmingham B15 2TH, United Kingdom

## Abstract

**Context:**

Alström syndrome is characterized by increased risk of cardiovascular disease from childhood.

**Objective:**

To explore the association between risk factors for cardiovascular disease, aortic pulse wave velocity, and vascular events in Alström syndrome.

**Design:**

Cross-sectional analyses with 5-year follow-up.

**Setting:**

The UK NHS nationally commissioned specialist clinics for Alström syndrome.

**Patients:**

Thirty-one Alström patients undertook vascular risk assessment, cardiac studies, and aortic pulse wave velocity measurement. Subsequent clinical outcomes were recorded.

**Interventions:**

Insulin resistance was treated with lifestyle intervention and metformin, and diabetes with the addition of glitazones, glucagon-like peptide 1 agonists, and/or insulin. Thyroid and T deficiencies were corrected. Dyslipidemia was treated with statins and nicotinic acid derivatives. Cardiomyopathy was treated with standard therapy as required.

**Main Outcome Measures:**

The associations of age, gender, and risk factors for cardiovascular disease with aortic pulse wave velocity were assessed and correlated with the effects of reduction in left ventricular function. Vascular events were monitored for 5 years.

**Results:**

Aortic pulse wave velocity was positively associated with the duration of diabetes (*P* = .001) and inversely with left ventricular ejection fraction (*P* = .036). Five of the cohort with cardiovascular events had higher aortic pulse wave velocity (*P* = .0247), and all had long duration of diabetes.

**Conclusions:**

Duration of diabetes predicted aortic pulse wave velocity in Alström syndrome, which in turn predicted cardiovascular events. This offers hope of secondary prevention because type 2 diabetes can be delayed or reversed by lifestyle interventions.

Alström syndrome is a rare (1 per million; OMIM 203800) autosomal recessive condition characterized by childhood-onset cone rod retinal dystrophy, neuronal hearing loss, obesity, and insulin resistance (1). The original phenotype described by Alström et al (1) in 1959 has been extended to include cardiomyopathy, type 2 diabetes, and renal and hepatic dysfunction (2–7). Subtle vascular lesions have been shown by brain magnetic resonance image scanning in Alström syndrome patients more than 30 years of age (8). The causative gene, *ALMS1*, has been identified (9, 10), and the ALMS1 protein localizes to the centrosome that is part of the intracellular ciliary apparatus in all eukaryotic cells. This has led to the classification of Alström syndrome as a ciliopathy (11–13). Alström syndrome is characterized by a cluster of potent causes for cardiovascular disease including insulin resistance, type 2 diabetes, dyslipidemia, renal dysfunction, and hypertension (7, 14, 15). Two adolescent clinical cohort studies have shown differing incidence of type 2 diabetes possibly related to obesity and also progressive age-dependent β-cell failure (16, 17). A further case series has demonstrated that hyperglycemia can be ameliorated by exercise and that hyperglycemia, dyslipidemia, hypertension, and hepatic steatosis can all be modified by lifestyle changes (18). Coronary artery disease has been described in the syndrome (19). Greater awareness of genetic causes of type 2 diabetes has led to identification of older subjects with Alström syndrome (20). There is therefore a clinical need to monitor vascular function in Alström subjects with appropriate risk factor intervention.

Elevated central pulse pressure measured as aortic pulse wave velocity (PWV) is indicative of large artery stiffening. Meta-analysis of outcome studies has confirmed that there is a 2-fold increase in all-cause and cardiovascular mortality in those with the highest tertile compared with the lowest (21). This effect is accentuated in patients with excess cardiovascular risk. Measurement of aortic PWV in the syndrome where multiple vascular risk factors are present from childhood is therefore of interest. In nonsyndromic obesity and diabetes, increased aortic PWV is linked with age and insulin resistance (22, 23). Aortic PWV has also been shown to independently predict cardiovascular mortality in diabetes, although serum lipids, renal function, and glycated hemoglobin (HbA1c) were not measured in the study (24). In addition, we have previously demonstrated elevated central artery pressure, independent of brachial artery pressure with high normal aortic PWV in children and young adults with Alström syndrome (25).

The present cross-sectional study has included a larger number and wider age range of Alström syndrome subjects. This report also explores the relationships between risk factors for cardiovascular disease including duration of diabetes as well as left ventricular function and aortic PWV in the syndrome. Cardiovascular events occurring during the poststudy period have been recorded.

## Subjects and Methods

### Ethical approval

All subjects gave written informed consent and were included in the DAS study (Defining the phenotype in Alström Syndrome) (UKCRN 9044, REC approval 10/H0203/33) funded by a UK Science Lottery Grant in association with Alström UK.

### Patients

Twenty-two Alström subjects attended UK National Health Service (NHS) nationally commissioned specialist clinics for Alström syndrome at Torbay Hospital between 2005 and 2010. The other nine were reviewed and tested by the Torbay team at Alström clinics in Leeds, Nottingham, Cardiff, and Truro. Eleven of the 31 subjects were female, and ages ranged from 11–44 years at the time of the baseline aortic PWV measurement.

### Clinical features

All subjects in the study had cone rod retinal dystrophy, acanthosis nigricans, obesity, and insulin resistance. Dyslipidemia characteristic of insulin resistance was present at the time of aortic PWV measurement in 28 of the 31 subjects, serum high-density lipoprotein (HDL) cholesterol was < 1.1 mmol/L, and/or serum triglycerides were > 2.0 mmol/L. All but one patient had preserved intelligence. Patients were screened annually to identify secondary hypothyroidism and, in males, T deficiency. Hormone deficiencies were corrected for at least 1 year before the vascular investigations (Table 1). Long-term statin therapy was prescribed for all patients > 25 years of age, but myositis developed in subject no. 22, necessitating withdrawal of treatment. Severe hypertriglyceridemia and renal impairment are frequent in Alström syndrome after adolescence. Nicotinic acid derivatives were therefore prescribed rather than fibrates because of their greater triglyceride-lowering effect and because of the risk of fibrate-induced myositis (14, 15).

**Table 1.** Demographics, Mutation Analysis, and Treatment of 31 Alström Subjects

Subject ID	Age, y	Ethnicity	Gender	Mutation 1	Mutation 2	DM	Treatment
1	27	White British	F	c.10775delC	c.10992G>A	Y	S, A, Th, Ins, NA
2	36	White British	M	c.1735delA	c.10483C>T	Y	S, A, Th, Ins, NA, IA, Test
3	38	White British	M	c.10775delC	c.11414delG	Y	S, A, Ins, Test
4	23	White British	F	c.10775delC	7534C>T	Y	A, Ins, Met
5	25	White British	F	c1735delA	c. 10483C>T	Y	A, Met, IA, Th
6	20	Mixed race	F	2225_2226dupA	c.1069C>T	N	Met, Th
7	17	White British	F	6829 C>T	9541C>T	N	Met, Th, PPI
8	20	White British	M	6829 C>T	9541C>T	N	Met
9	44	White British	M	c.10483C>T	c.10775delC	Y	Met, IA, S, Test
10	38	White British	F	c.10775delC	c.1131C>T	Y	Met
11	35	White British	M	7132_7133insA	7132_7133insA	N	HD
12	39	White British	M	c.9001C>T	c.9001C>T	Y	Met, IA, Test, Th, A, S
13	17	White British	F	6590delA	c.8832C>G	Y	Ins
14	21	White British	M	NA	NA	Y	β, Ins, Met
15	15	White British	M	c.10775delC	c.5362A>G	Y	Met
16	20	White British	M	c.6895delG	c.10483C>T	Y	Met
17	19	White British	M	c.6526C>T	c.11107C>T	N	Ins, Met
18	12	White British	M	c.6526C>T	c.11107C>T	Y	Met
19	20	White British	F	c4026delA	c.6325G>T	N	PPI
20	11	White British	M	c.10885C>T	c.11107C>T	N	Test
21	12	White British	M	NA	NA	N	Met
22	34	White British	M	c.10775delC	10483C>T	Y	Ins, β, PPI, HD
23	17	Pakistani	F	7543 + 895del1444	c1110del	Y	Met
24	19	Pakistani	F	7543 + 895del1444	c1110del	Y	Met, Ins
25	11	Pakistani	M	7543 + 895del1445	c1110del	N	Met
26	15	Pakistani	M	7543 + 895del1446	c1110del	Y	Met
27	12	Pakistani	M	7543 + 895del1447	c1110del	N	Met
28	15	Pakistani	F	c.5081delC	c.5081delC	Y	Met
29	35	White British	F	c.10483C>T	6305C>A	N	S, A, Th, Diuretic
30	38	White British	M	c.10483C>T	6305C>A	N	S, A, Th
31	31	White British	M	c10483C>T	c9541C>T	N	S, A, Th

Abbreviations: DM, diabetes mellitus; S, statin; A, angiotensin-converting enzyme inhibitor or A2 blocker; Met, metformin; Ins, insulin; IA, incretin analog; Th, thyroxine; PPI, proton pump inhibitor; Test, testosterone; HD, hemodialysis; NA, mutation confirmed but not available; M, male; F, female; Y, yes; N, no; β, beta-blocker. Genetics: Subjects 7/8 and 23/24 are sibling pairs; subjects 25 to 27 are from a consanguineous family; subjects 13 (c.8832C>G) and 15 (c.5362A>G) have missense mutations of uncertain significance; subject 19 has a c.6325G>T polymorphism.

### Genetic confirmation of diagnosis

Twenty-nine subjects in the study were shown to have two pathological mutations in *ALMS1* (Table 1). In the other two, genetic confirmation had been reported, but the results were not available. Both of these presented with classical clinical features of acanthosis nigricans, childhood-onset cone rod retinal degeneration, neuronal deafness, early onset obesity, insulin resistance, and cardiomyopathy with preserved intelligence.

### Blood pressure measurement

Blood pressure was recorded after a 10-minute rest at the brachial artery of the dominant arm using a validated semiautomated oscillometric device in the seated position. All hemodynamic measurements were recorded in a quiet, temperature-controlled room.

### Aortic PWV

Assessment of aortic PWV is the “gold standard” procedure and the validated method for assessing arterial stiffness (21). In brief, aortic PWV was measured as the time taken for the pulse to travel from a three-lead electrocardiogram (ECG)-gated signal to the first upstroke of the pulse wave between the carotid and femoral sites. PWV was measured using the SphygmoCor system (Atcor Medical). All measurements were performed by a single experienced operator, trained at a well-established vascular laboratory. Previous studies undertaken to assess reproducibility in this laboratory have demonstrated a within-observer difference for repeated measures of aortic PWV (recorded by SphygmoCor) of 0.07 ± 0.24 m/s (mean ± SEM) within a patient coefficient of variation of 3% (25).

### Echocardiography

In 27 of the subjects, transthoracic echocardiography was performed in all four clinical centers by experienced echocardiographers and a cardiologist familiar with Alström subjects on the same day as the aortic PWV tests. In the other four subjects, echocardiography was undertaken within 6 months of aortic PWV. Left ventricular ejection fraction was measured by biplane or single plane method of disks (modified Simpson's rule) (26). Left ventricular ejection fraction was measured in all of the subjects by this method to standardize comparison between cardiac function and aortic PWV.

### Cardiac magnetic resonance imaging

Cardiac magnetic resonance imaging (CMR) was performed on 17 of the adult subjects (1.5T Avanto; Siemens Healthcare). Left and right ventricular volumes, mitral annular plane systolic excursion, ejection fraction, and left ventricular mass index were acquired in line with standard CMR protocols. Left atrial volumes were calculated using the biplane area-length method. The four-chamber and short axis steady-state free precession cine images (ECG-gated, True-FISP; temporal resolution, 40–50 ms; TR, 3.2 ms; TE, 1.6 ms; −FA, 60°; slice thickness, 7 mm) at the mid left ventricle (papillary muscle) were used for myocardial deformation analysis. Late gadolinium enhancement imaging was performed 7–10 minutes after 0.15 mmol/kg of gadolinium contrast bolus (Gadovist; Bayer Health Care). All patients consented to receive iv gadolinium 0.15 mmol/kg (www.scmr.org protocols). CMR was used to monitor myocardial fibrosis and to detect the occurrence of changes consistent with infarction. It was not considered ethically acceptable to perform CMR routinely on younger deaf blind Alström subjects, and therefore left ventricular ejection fraction was measured from echocardiography as above in all subjects.

### Biochemical tests

Serum C-peptide and/or serum insulin levels were measured to assess insulin secretion (Endocrinology Unit, level D, South Block, Southampton General Hospital). Samples were processed and deep-frozen for later assay within 2 hours of venesection. Serum lipids, renal function, blood glucose (fluoride), and HbA1c levels were measured by standard laboratory procedures. Insulin resistance was measured as postprandial serum C-peptide/glucose ratio or fasting serum insulin level (27, 28).

### Clinically significant cardiovascular events

Clinically significant events were confirmed by angina-like symptoms leading to confirmatory coronary angiography (two cases), autopsy after cardiac-related death (two cases), duplex arterial scans and serial CMR (one case), and carotid plaque (one case). The other patients had no symptoms suggestive of angina or evidence of myocardial infarction on serial electrocardiography or CMR. Patients over 20 years of age designated as free from cardiovascular disease had normal lower limb arterial duplex scans and an absence of carotid artery plaques.

### Statistics

Associations between aortic PWV and the following variables was sought: age, gender, body mass index (BMI), duration of diabetes mellitus, HbA1c, log serum creatinine, mean systemic arterial blood pressure, serum triglycerides, serum cholesterol, serum HDL cholesterol, non-HDL cholesterol, and left ventricular ejection fraction. The variables were then interacted against aortic PWV in a general linear model, with log transformation where appropriate to achieve conformity of Gaussian distribution. Insignificant terms were removed in a stepwise fashion using an F test (see Supplemental Table 1). All statistics were carried out in the software program “R” (http://www.r-project.org/).

The analysis was repeated for development of cardiovascular disease during follow-up, including aortic PWV as a variable.

## Results

**Figure 1.**
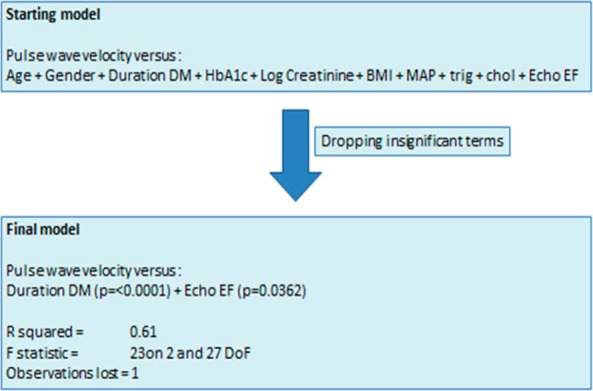
Linear regression model of risk factors associated with arterial PWV in 31 Alström subjects. DM, diabetes mellitus; MAP, mean arterial pressure mmHg; trig, serum triglycerides; Chol, serum non-HDL cholesterol; Echo EF, echocardiogram left ventricular ejection fraction.

**Figure 2.**
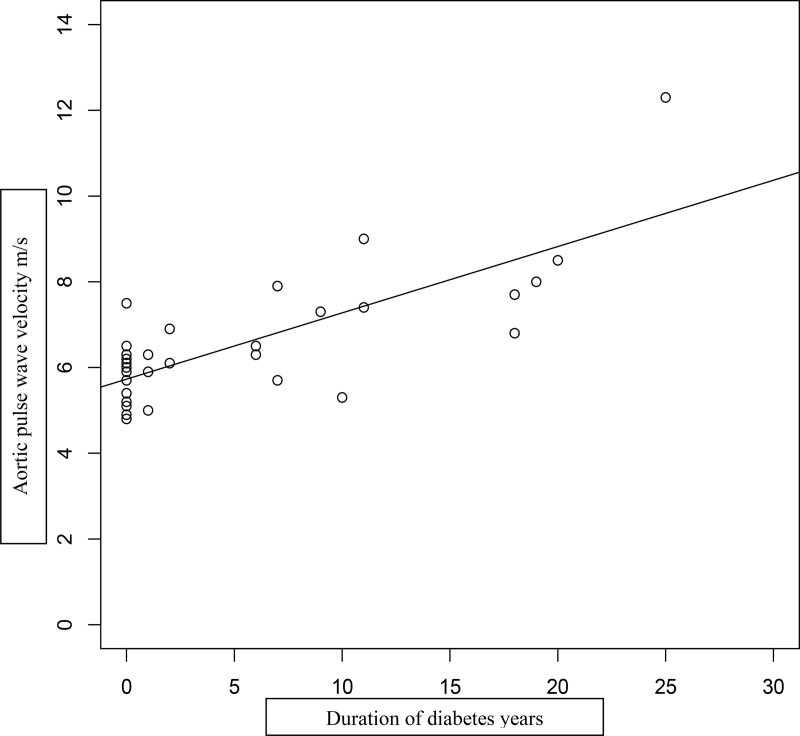
Correlation between aortic PWV and duration of diabetes in 31 Alström subjects. R^2^ = 0.564.

**Figure 3.**
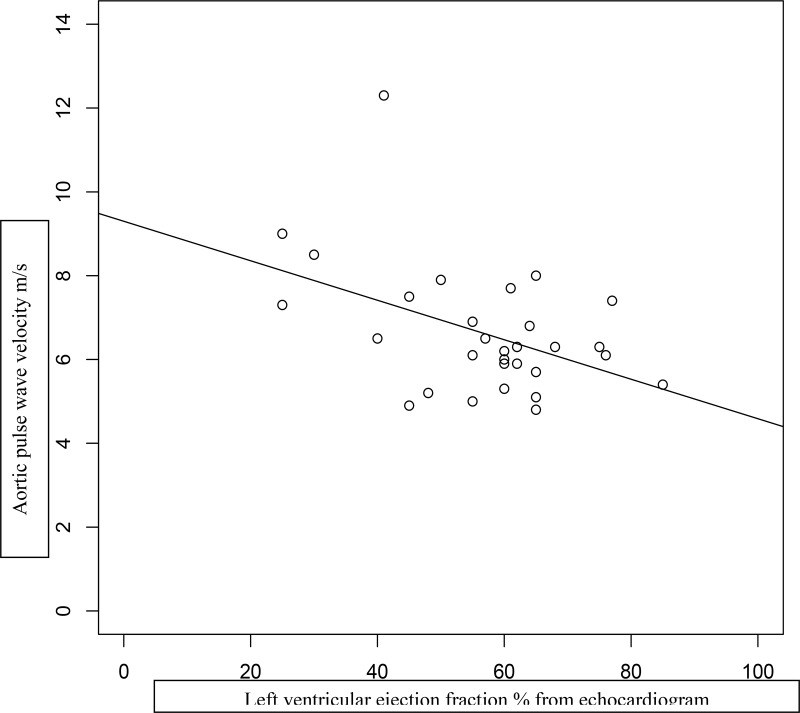
Inverse correlation of aortic PWV vs left ventricular ejection fraction in 31 Alström subjects. R^2^ = 0.172.

Demographic, genetic, anthropometric, and clinical data are shown in Table 1. Biochemical and physiological data are shown in Table 2. Severe insulin resistance is confirmed biochemically in 26 of the subjects by fasting serum insulin (normal range, <150 pmol/L) (28) or postprandial C-peptide/glucose ratio (27). The five subjects for whom biochemical confirmation is not available all had truncal obesity and acanthosis nigricans. Table 3 shows the PWV values at baseline and evidence of cardiovascular disease during follow-up. Figure 1 shows the starting characteristics of associations with PWV in a general linear model before removal of nonsignificant terms. After logistic regression, duration of diabetes was positively related to PWV (*P* = .001), and left ventricular ejection fraction was negatively and independently associated (*P* = .036) (R^2^ = 0.61). Figures 2 and 3, Supplemental Figure 1, and Supplemental Table 1 illustrate the relationships between these variables.

**Table 2.** BMI and Biochemical Data: 31 Alström Subjects Ordered by Gender, Age, and Diabetes Duration

Subject ID	Sex and Age, y	DM Duration, y	BMI, kg/m^2^	Serum Cholesterol, mmol/L (mg/dL)	HDL Cholesterol, mmol/L (mg/dL)	Non-HDL Cholesterol, mmol/L (mg/dL)	Serum Triglycerides, mmol/L (mg/dL)	EGFR, mL/min	Fasting Insulin, pmol/L (IU/mL)	C-peptide/Glucose, pmol/mmol (ng/mL/mg/dL)	HbA1c, mmol/mol (%)	UMA, mg/mmol (mg/g)
With DM												
10	F 38	7	39	4.8 (185.8)	1.28 (49.5)	3.5 (136.2)	1.3 (115)	95		750 (0.13)	50 (6.7)	
1	F 27	11	27.4	5.9 (228.3)	0.66 (25.5)	5.2 (202.8)	5.7 (503)	10		652 (0.11)	49 (6.6)	7.1 (62.8)
5	F 25	11	28	7.4 (286.4)	1.33 (51.5)	6.1 (234.9)	2.6 (230)	90		215 (0.04)	65 (8.1)	4.5 (39.8)
4	F 23	9	31.6	4.8 (185.8)	0.9 (34.8)	3.9 (150.9)	4.2 (372)	65		195 (0.03)	94 (10.9)	0.1 (0.9)
24	F 19	10	32.1	5.5 (212.9)	0.8 (31.0)	4.7 (181.9)	2.4 (213)	54		NA	83 (9.8)	56.1 (495.9)
13	F 17	6	26	5.1 (197.4)	0.74 (28.6)	4.4 (168.7)	2.5 (222)	95		130 (0.02)	86 (10.1)	1.4 (12.4)
23	F 17	1	26.9	2.4 (92.9)	0.6 (23.2)	1.8 (69.7)	3.1 (275)	65		NA	48 (6.5)	1.56 (13.8)
9	M 44	18	32	3.1 (120.0)	0.53 (20.5)	2.6 (99.5)	4.7 (416)	63		1159 (0.19)	31 (5.0)	26.1 (230.7)
12	M 39	18	42.8	4.4 (170.3)	1.64 (63.5)	2.8 (106.8)	1.1 (97)	75		498 (0.08)	61 (7.7)	1.7 (15.0)
3	M 38	25	27	4.3 (166.4)	0.93 (36.0)	3.4 (130.4)	4.2 (372)	14		162 (0.03)	104 (11.7)	178.7 (1579.7)
2	M 36	19	36.4	7.5 (290.3)	1.06 (41.0)	6.4 (249.2)	13.0 (1152)	52		229 (0.04)	84 (9.8)	6.3 (55.7)
22	M 34	20	23	2.2 (85.1)	0.86 (33.3)	1.3 (51.9)	3.0 (266)	15		362 (0.06)	56 (7.3)	1000.0 (8840.0)
31	M 31	2	33	3.6 (139.3)	0.8 (31.0)	2.8 (108.4)	1.6 (142)	75		435 (0.07)	44 (6.2)	24.0 (212.2)
14	M 21	6	24.6	6.3 (243.8)	0.77 (29.8)	5.5 (214.0)	8.8 (780)	95		177 (0.03)	77 (9.2)	2.6 (23.0)
16	M 20	7	33	5.7 (220.6)	0.83 (32.1)	4.9 (188.5)	2.7 (240)	95		886 (0.15)	30 (4.9)	29.0 (256.4)
8	M 20	2	24.8	5.1 (197.4)	0.71 (27.5)	4.4 (169.9)	3.0 (264)	95		828 (0.14)	34 (5.3)	2.6 (23.0)
18	M 18	1	30.4	4.1 (158.7)	0.63 (24.4)	3.5 (134.3)	3.9 (346)	90		128 (0.02)	54 (7.1)	0.4 (3.5)
26	M 15	1	24.7	4.4 (170.3)	1.0 (38.7)	3.4 (131.6)	3.3 (292)	124		NA	72 (8.7)	1.5 (13.3)
Mean [median]	26.8	9.7	30.2	4.8 (186.2)	0.9 (34.6)	3.9 (151.6)	[3.1 (270)]	70.4		454 (0.08)	62.33 (7.87)	[4.5 (39.8)]
SD [IQR]	9.3	7.5	5.4	1.5 (57.0)	0.3 (11.0)	1.4 (54.5)	[2.5 (223)]	31.9		328 (0.06)	22 (2)	[1.5 (13.8)]
Without DM												
29	F 35	0	28.7	5.5 (212.9)	0.8 (31.0)	4.7 (181.9)	5.7 (505)	80	150 (21.6)		5.5 (37)	[6.2 (54.8)]
6	F 20	0	29	4.7 (181.9)	1.07 (41.4)	3.6 (140.5)	3.1 (277)	95		1175 (0.20)	26 (4.5)	5.2 (46.0)
19	F 20	0	36.3	5.6 (216.7)	1.26 (48.8)	4.3 (168.0)	2.1 (186)	52	253 (36.4)		37 (5.5)	
7	F 17	0	25	5.3 (205.1)	0.75 (29.0)	4.6 (176.1)	3.6 (322)	90		751 (0.13)	31 (5.0)	17.3 (152.9)
28	F 15	0	35					90		NA	42 (6.0)	
30	M 38	0	32	5.4 (209.0)	0.9 (34.8)	4.5 (174.2)	5.9 (523)	85	211.5 (30.4)		36 (5.4)	4.1 (36.2)
11	M 35	0	29	4.9 (189.6)	1.09 (42.2)	3.8 (147.4)	6.3 (558)	26		566 (0.09)	32 (5.1)	6.2 (54.8)
17	M 19	0	28.9	4.9 (189.6)	0.62 (24.0)	4.3 (165.6)	3.5 (310)	90		1022 (0.17)	31 (4.9)	0.4 (3.5)
15	M 15	0	26	3.0 (116.1)	0.7 (27.1)	2.3 (89.0)	1.2 (106)	95	165 (23.7)		44 (6.2)	4.9 (43.3)
21	M 12	0	27.3	5.5 (212.9)	0.7 (27.1)	4.8 (185.8)	4.0 (354)	90		NA		
27	M 12	0	27.3	3.7 (143.2)	0.7 (27.1)	3.0 (116.1)	4.0 (354)	90		NA	37 (5.5)	1.6 (14.1)
20	M 11	0	33.4	5.9 (228.3)	0.57 (22.1)	5.3 (206.3)	3.4 (301)	90		314 (0.05)	40 (5.8)	1.7 (15.0)
25	M 11	0	25.6	4.9 (189.6)	0.9 (34.8)	4.0 (154.8)	3.1 (275)	90	530 (76.3)		36 (5.4)	1.1 (9.7)
Mean [median]	20.0		29.5	4.9 (191.2)	0.8 (32.4)	4.1 (158.8)	[3.8 (339)]	81.8	262 (37.7)	765.6 (0.1)	37.8 (5.6)	[4.9 (43.1)]
SD [IQR]	9.7		3.6	0.8 (32.4)	0.2 (8.2)	0.8 (32.1)	[1.5 (134.1)]	20.1	155.2 (22.3)	345.5 (0.1)	8.8 (0.8)	[1.6 (14.1)]
*P* value	.06		.69	.77	.55	.65	.89	.23			.0003	.27

Abbreviations: DM, diabetes mellitus; F, female; M, male; EGFR, estimated glomerular filtration rate; IQR, interquartile range. *P* value represents unpaired *t* test diabetic vs nondiabetic subjects.

**Table 3.** Characteristics and Outcome in 31 Alström Subjects Ordered by Gender, Age, and Diabetes Duration

Subject ID	Sex and Age, y	DM Duration, y	MAP, mm Hg	PWV, m/s	Arterial Duplex Scan	Carotid Scan	Coronary Artery Disease Assessment
With DM							
10	F 38	7	80	7.9	Normal	No atheroma	Sudden post-operative death >50% narrowing LAD at autopsy
1	F 27	11	115	9	Normal	No atheroma	Acute coronary syndrome PCI
5	F 25	11	101	7.4	Normal	No atheroma	CMR scan-no infarction
4	F 23	9	76	7.3	Normal	No atheroma	CMR scan-no infarction
24	F 19	10	92	5.3	Normal	No atheroma	No infarction on ECG or echocardiogram
13	F 17	6	86	6.5	Normal	No atheroma	CMR scan-no infarction
23	F 17	1	98.5	5	Normal	No atheroma	No infarction on ECG or echocardiogram
9	M 44	18	116	6.8	Iliac atheroma	No atheroma	CT coronary calcification. CMR scan myocardial infarction
12	M 39	18	101	7.7	Normal	No atheroma	CMR scan-no infarction
3	M 38	25	120	12.3	Iliac atheroma	No atheroma	Acute coronary syndrome PCI
2	M 36	19	101	8	Normal	Plaque	Occlusion of RCA at autopsy
22	M 34	20	99	8.5	Iliac atheroma	No atheroma	Acute coronary syndrome angiogram normal
31	M 31	2	125	6.9	Normal	No atheroma	No infarction on ECG or echocardiogram
14	M 21	6	90	6.3	Normal	No atheroma	No infarction on ECG or echocardiogram
16	M 20	7	101.5	5.7	Normal	No atheroma	CMR scan-no infarction
8	M 20	2	97	6.1	Normal	No atheroma	CMR scan-no infarction
18	M 18	1	112.5	6.3	Normal	No atheroma	CMR scan-no infarction
26	M 15	1	97.5	5.9	—	No atheroma	No infarction on ECG or echocardiogram
Mean	F 7/18 26.8	9.7	100.5	7.2			
SD	9.3	7.5	13.3	1.7			
Without DM							
29	F 35	0	116	6.5	Normal	No atheroma	No infarction on ECG or echocardiogram
6	F 20	0	94	5.1	Normal	No atheroma	CMR scan-no infarction
19	F 20	0	119	6.2	—	No atheroma	CMR scan-no infarction
7	F 17	0	84	6.3	Normal	No atheroma	CMR scan-no infarction
28	F 15	0	122.5	6.1	—	No atheroma	No infarction on ECG or echocardiogram
30	M 38	0	100.5	7.5	Normal	No atheroma	No infarction on ECG or echocardiogram
11	M 35	0	62	4.9	Normal	No atheroma	CMR scan-no infarction
17	M 19	0	98	5.4	—	No atheroma	CMR scan-no infarction
15	M 15	0	92	6	Normal	No atheroma	CMR scan-no infarction
21	M 12	0	90.5	5.7	—	No atheroma	CMR scan-no infarction
27	M 12	0	80	5.2	—	No atheroma	No infarction on ECG or echocardiogram
20	M 11	0	93.5	5.9	—	No atheroma	CMR scan-no infarction
Mean	F 5/13 20	0	97.5	5.8			
SD	9.7		17.3	0.8			
*P* value	.061		.602	.006			

Abbreviations: F, female; M, male; DM, diabetes mellitus; MAP, mean arterial pressure; PCI, percutaneous coronary intervention with stenting. *P* value represents unpaired *t* test diabetic vs nondiabetic subjects.

Five of the 12 subjects over the age of 25 years (ID no. 1, 2, 3, 9, and 10), all of whom had long-standing type 2 diabetes, developed clinically significant cardiovascular disease. Logistic regression in the general linear model was also applied to the occurrence of cardiovascular disease, which showed that only aortic PWV was independently associated with cardiovascular disease (*P* = .0247; Figure 1 and Table 1). The full description of relationships between aortic PWV, duration of diabetes, gender, non-HDL cholesterol, and age is shown in Supplemental Figure 2.

Seven subjects over 25 years of age at the time of the baseline assessment of aortic PWV have remained free from clinically significant cardiovascular disease. Five have maintained normal HbA1c levels, one of whom (ID no. 11) was treated with hemodialysis for end-stage renal failure. Another subject (ID no. 31) received renal and whole pancreas transplantation 10 and 9 years before the baseline study and remains well 5 years later. One subject (ID no. 22) with long-standing type 2 diabetes had concurrent severe cirrhosis of the liver due to nonalcoholic liver disease in association with the syndrome. He had maintained low systemic arterial blood pressure, serum cholesterol < 3 mmol/L, intermediate aortic PWV 8.2 m/s, but relative freedom from cardiovascular disease verified by normal coronary arteriogram. He also had normal carotid ultrasound scans and minor disease on lower limb arterial duplex scans (15% stenosis of the right iliac artery).

## Discussion

The very early occurrence of severe insulin resistance, dyslipidemia, hypertension, and progression to type 2 diabetes implies high risk for cardiovascular disease. The current case series demonstrates that aortic PWV, a highly significant determinant of future vascular events, is increased in Alström patients in direct relation to the duration of diabetes, and that this relationship is independent of age. It is well established that aortic PWV is strongly linked with advancing age in the general population (22, 23, 29, 30). The increase is most marked from the fifth decade onward. The young age range of our Alström subjects may explain why age was not strongly associated with aortic PWV in this study. Conversely, aortic PWV may be influenced by cardiac function, and in particular left ventricular ejection fraction. The relationship is complex because severely compensated left ventricular dysfunction with left ventricular ejection fraction of 24% has been shown to be associated with lower aortic PWV (31). During episodes of decompensation in patients with less severe heart failure, aortic PWV has been shown to increase (30). In keeping with these findings, the relationship between diabetes duration and aortic PWV in our case series is modified by left ventricular ejection fraction (Supplemental Figure 1). Five of 12 cases in the series over 25 years of age developed coronary artery disease (two myocardial infarction, two acute coronary syndrome requiring stenting, and one a silent myocardial infarct demonstrated by serial CMR). Previous CMR studies in younger Alström syndrome subjects have shown variable diffuse fibrosis and dilated cardiomyopathy, but no infarction (32, 33). In the present case series, one subject showed clear changes of infarction on serial CMR scans. All of those with cardiovascular events had long duration of diabetes and high aortic PWV. These events occurred between the ages of 28 and 48 years, representing an extremely high risk of cardiovascular disease in this cohort of Alström subjects. Extensive normative data for aortic PWV have shown in nondiabetic populations that levels > 12 m/s indicate a striking risk of vascular disease, with similar findings in diabetic subjects (21, 24). Our younger group of Alström subjects rarely reached such high levels before development of cardiovascular disease. This may be explained by the intensity of multiple vascular risk factors from childhood in our cohort, which would be expected to accelerate the development of cardiovascular disease. Aortic PWV also predicts mortality in populations with chronic renal disease (34), although in our series the development of end-stage renal failure without diabetes was not associated with raised aortic PWV or cardiovascular disease. It may be relevant that renal dysfunction in Alström syndrome occurs as a result of diffuse fibrosis unrelated to diabetes (7, 35). Urinary microalbumin/creatinine ratios (UMAs) are often normal in the syndrome, and UMA ratio increased only modestly in subject no. 11 on hemodialysis but with normal glucose tolerance (Table 2). The six subjects with renal impairment and diabetes had modest proteinuria, with UMA levels ranging from 6.3 to 1000 μmol/mmol (mg/g). Although insulin resistance and dyslipidemia are genetically determined in Alström syndrome, there is evidence that development of type 2 diabetes may be delayed and the hyperlipidemia effectively treated (15). The published cohorts of Alström subjects followed through adolescence suggest that early onset of type 2 diabetes is determined by obesity (16, 17). Lifestyle modification has also been shown to be effective in influencing dyslipidemia, glucose intolerance, and hypertension (18). There is hope therefore that calorie restriction and exercise from childhood may not only delay the onset of diabetes but possibly prevent or delay cardiovascular disease in Alström patients. The preservation of vascular health in one subject (ID no. 31) with dual renal and pancreas transplantation and statin and hypotensive therapies until 36 years of age is also encouraging. Both transplants continued to function well, with normal HbA1c and serum creatinine at the time of the study and normal aortic PWV (6.9 m/s).

Four other subjects with severe insulin resistance but without progression to diabetes (age range, 35 to 38 y) have also remained free from overt cardiovascular disease (Table 3). A detailed study has shown that measures of vascular stiffness are increased in relation to severity of insulin resistance in nondiabetic individuals (36). Any such effect may be obscured in our patient group by the younger age range and dominant effect of diabetes duration.

One limitation of this study is the small number involved because of the rarity of Alström syndrome. The widespread organ fibrosis in Alström syndrome may have effects on both aortic PWV and cardiovascular disease not present in nonsyndromic metabolic syndrome and type 2 diabetes. With larger numbers of associations between aortic PWV and cardiovascular disease with renal failure, serial HbA1c levels, and non-HDL cholesterol levels, gender and age might have emerged. Its strengths lie in the coherence of the patient group, consistency of methodology, and completeness of follow-up. The insulin resistance of Alström syndrome does mirror the propensity for development of diabetes, arterial stiffness, and cardiovascular disease that is seen in the wider population (37, 38). As in the wider population, insulin resistance is accompanied by dyslipidemia, hypertension, and frequent but not inevitable progression to type 2 diabetes. Simple lifestyle intervention is effective in preventing progression of impaired glucose tolerance for up to 10 years in diabetes prevention programs (39–41). This can also be achieved in Alström patients despite the dual sensory loss. The effect on the development of cardiovascular disease remains to be determined.

In summary, we have studied vascular risk factors associated with aortic PWV and cardiovascular disease in a well-characterized group of subjects with a rare genetic insulin-resistant syndrome. The data are consistent with the hypothesis that arterial stiffness and cardiovascular disease are predominantly determined by the duration of diabetes in older subjects with the syndrome. Because the onset of diabetes can be delayed in Alström syndrome by lifestyle modification, there is a clear indication that every Alström family should be afforded the most appropriate access to nutritional advice and empowerment to exercise despite the dual sensory loss. It is also recommended that dyslipidemia in the syndrome should be treated from adolescence. Whether these tentative conclusions can be generalized to the growing number of obese insulin-resistant adolescents at high risk of type 2 diabetes is not known but is worthy of study.
